# Knowledge and Attitudes toward Genetic Testing for Autism Spectrum Disorders among Parents of Affected Children in Taiwan

**DOI:** 10.3390/genes13020239

**Published:** 2022-01-27

**Authors:** Ming Li, Shi-Xi Zhao, Wei-Ju Chen, Tse-Yang Huang, Lei-Shih Chen

**Affiliations:** 1 Department of Health Sciences, Towson University, Towson, MD 21252, USA; mli@towson.edu; 2 Department of Health, Exercise & Sports Sciences, University of New Mexico, Albuquerque, NM 87131, USA; shixizhao@unm.edu; 3 Department of Psychology, The University of Texas Permian Basin, Odessa, TX 79762, USA; chen_w@utpb.edu; 4 Department of Special Education, National Tsing Hua University, Hsinchu 30013, Taiwan; tseyanghuang@mx.nthu.edu.tw; 5 Department of Health and Kinesiology, Texas A&M University, College Station, TX 77843, USA

**Keywords:** autism spectrum disorders, genetic testing, attitudes, knowledge, Taiwan

## Abstract

The prevalence of autism spectrum disorders (ASD) in Taiwan has been increasing, and genetic testing for ASD has been available and provided to parents of children diagnosed with ASD in Taiwan. However, there is still limited understanding of Taiwanese parents’ knowledge of and attitudes toward such testing. Therefore, the present study addressed this gap by assessing the attitudes toward as well as actual and perceived knowledge of ASD genetic testing among Taiwanese parents of children diagnosed with ASD. A sample of 443 parents of children with ASD recruited from 236 public schools in Taiwan completed a paper-and-pencil survey. Although parents generally held favorable attitudes toward ASD genetic testing, they had deficient knowledge of such test (with only a 31.4% average correct rate on the actual knowledge scale). Tailored health education materials should be developed to improve the knowledge of ASD genetic testing among parents with affected children in Taiwan.

## 1. Introduction

Autism spectrum disorders (ASD) are characterized by deficits in social communication and interactions, as well as the presence of restricted, repetitive, and stereotyped patterns of behaviors and interests [1]. The worldwide prevalence of ASD was estimated to be one in 160 children (0.6%) [2]. In Taiwan, the reported prevalence of ASD is higher, ranging from 1.0% to 1.2%, based on three representative studies with large samples [3,4,5]. Due to the insufficient number of health professionals with expertise in ASD (particularly, child psychiatrists) [6,7], limited clinical infrastructure for ASD assessment and diagnosis [6,7], and parents’ reluctance to seek help due to the cultural stigmatization associated with mental disorders [8,9], the actual prevalence of children with ASD in Taiwan could be higher. Moreover, ASD has become one of the fastest-growing developmental disorders in Taiwan since 2011 [10].

Genetics is an important risk factor for ASD, with an estimated heritability of 80% based on a recent study with a sample of two million people across several countries [11]. Over one hundred ASD susceptibility genes [12,13] have been identified that could contribute to the development of ASD, including copy number variants (CNVs) (e.g., deletions of 16p11.2, duplications of 15q11.2) [13,14,15], de novo mutations (e.g., CHD8, DEAF1) [12,13], and certain syndromic disease genes that are associated with ASD (e.g., FMR1 gene in Fragile X) [13]. With the advancement of medical biotechnology in the past two decades, several clinical genetic testing methods have been used for the genetic diagnosis of ASD [16,17,18,19]. For instance, chromosomal microarray analysis (CMA) or high-resolution array comparative genomic hybridization (array CGH) can detect certain CNVs that are associated with ASD [17,18]. Fragile X testing can detect the expansions of the FMR1 gene, which could not be identified through CMA [20]. Whole-exome sequencing is also available for children with ASD to identify inherited causes of ASD [12,19].

The current medical guideline for genetic diagnosis of ASD recommends both CMA and Fragile X testing as the first-tier genetic testing for ASD [17,18] as such testing can potentially benefit the families of children with ASD. For example, genetic testing for ASD could: (1) assist in detecting the genetic cause of ASD and reduce the diagnosis odyssey for parents [18,21]; (2) provide personalized treatment and management for children with ASD [17,18,22]; and (3) help families make informed reproductive decisions by providing genetic risk evaluation information [18,23,24]. Due to these benefits, several major specialty organizations (e.g., American Academy of Pediatrics [22,23,24], American Academy of Neurology [25,26], and American College of Medical Genetics and Genomics [18]) have recommended genetic testing for all children diagnosed with ASD.

Research has shown that parents’ intention to pursue genetic testing for their children is significantly associated with their knowledge of ASD genetic testing and their attitudes toward ASD genetic testing [27]. However, previous studies conducted in the United States and Europe have demonstrated that although parents of children with ASD generally hold favorable attitudes toward genetic testing for ASD, they have deficient knowledge and awareness of such testing [27,28,29,30,31]. Furthermore, many parents of children with ASD may have unrealistic expectations, misperceptions, or misinformation regarding genetic testing for ASD [32,33].

While ASD genetic testing has been available in Taiwan for many years [34,35], there is still limited research on parents’ attitudes and knowledge regarding ASD genetic testing in Taiwan. It is important to understand Taiwanese parents’ knowledge and attitudes toward ASD genetic testing. Without this critical information before offering such testing, physicians or other health professionals in Taiwan may not be able to provide tailored counseling, education, and support services, address parents’ potential concerns regarding genetic testing for ASD, and assist parents in making informed decisions. To fill this gap, the present study examined the knowledge and attitudes regarding ASD genetic testing among parents of children with ASD in Taiwan. Specifically, this study seeks to address the following research questions:

(1) What is the level of knowledge of genetic testing for ASD among Taiwanese parents of children with ASD?
(2) What are the attitudes toward ASD genetic testing among parents of affected children in Taiwan?
(3) What are the factors associated with these parents’ knowledge of and attitudes toward genetic testing for ASD?

## 2. Materials and Methods

### 2.1. Participants and Study Design

This study was part of a larger study designed to examine views regarding genetic testing among parents of children with ASD in Taiwan [35,36,37]. The research team retrieved a list of all public elementary schools with special education classes in Hsinchu City and County, Taoyuan County, and Miaoli County from the official website of the Department of Education in Taiwan. The research team then made phone calls to the special education teachers in these schools to obtain the exact number of children with ASD in their schools as well as to ask the teachers to help with the recruitment for this study. Parents of all the children with ASD enrolled in these schools were directly contacted by their special education teachers and invited to participate in this study. Subsequently, a package containing the survey and information sheet was distributed to all potential participants. To increase the sample size, the research team also expanded the recruitment to other areas, including Taipei City, New Taipei City, Taichung City, Tainan City, Kaohsiung City, Yilan County, Chiayi City, Chiayi County, and Yunlin County. A total of 236 schools participated in this study; among the 862 surveys that were sent out, 451 surveys were returned yielding a response rate of 52.3%. The final sample size was 443 after removing incomplete and duplicated surveys. All research protocols were approved by the Institutional Review Board at Texas A&M University.

### 2.2. Measurement

A paper-and-pencil survey was developed based on previous literature [36,37,38,39,40,41,42] and past qualitative data collected by the research team [28,43,44,45,46]. The survey was reviewed by a panel of experts in the fields of medicine, bioethics, pediatrics, special education, as well as social and behavioral science, and it was revised based on their feedback. The survey was then further revised based on the eight cognitive interviews, five retrospective interviews, and pilot tests with parents of children with ASD conducted by the research team. The survey was administered in traditional Chinese, which is the official language of Taiwan, for the convenience of the participants. For the purpose of this study, we extracted the following sections from the large survey: (1) parents’ actual and perceived knowledge of genetic testing for ASD; (2) parents’ attitudes toward ASD genetic testing; and (3) other variables including the parents’ sociodemographic characteristics, information regarding the children’s ASD diagnosis, and family history of ASD.

Actual knowledge of ASD genetic testing: Parents’ actual knowledge was measured using seven multiple-choice questions. One point was assigned for each correct answer to compute an overall knowledge score. The theoretical score for actual knowledge is 0–7.

Perceived knowledge of ASD genetic testing: To measure parents’ perceived knowledge of ASD genetic testing, we used a single item, “how much do you know about ASD genetic testing?” rated on a 5-point Likert-type scale ranging from very little, a little, average, a lot, to a great deal.

Attitudes toward ASD genetic testing: A 5-item scale with a 4-point Likert-type rating ranging from 1 (strongly disagree) to 4 (strongly agree) was used to measure parental attitudes toward ASD genetic testing (e.g., “all children diagnosed with ASD should undergo ASD genetic testing”).

Attitudes toward the cost of ASD genetic testing: To examine attitudes regarding the cost of ASD genetic testing, we used the item, “Undergoing ASD genetic testing is costly” rated on a 4-point Likert-type scale ranging from 1 (strongly disagree) to 4 (strongly agree). In addition, we asked parents who they think should pay for ASD genetic testing with three options including national health insurance, self-pay, and other insurance. Parents were asked to fill out a dollar amount in the following question, “Suppose you had to pay out-of-pocket for undergoing ASD genetic testing, what is an acceptable amount for you? NTD_______.”

Other variables: Sociodemographic variables (e.g., participant’s age, gender, annual household income, educational level, marital status, and employment status) and information regarding the numbers of children with ASD, type of ASD, severity level of ASD, and parents’ family history of ASD were also collected in the survey.

### 2.3. Data Analysis

Descriptive statistics were used to examine the central tendency, variability, percentages, and frequency distribution of the variables. Psychometric testing (i.e., reliability using Cronbach’s alpha and validity using confirmatory factor analysis) was conducted for the construct of attitudes. Bivariate correlations were also conducted to assess the parental attitudes and knowledge’ associations with the continuous variables (i.e., participant’s age, annual household income, numbers of children in the family, numbers of children with ASD, and severity level of ASD-affected child) and binary variables (i.e., participants’ gender, education status, marital status, employment status, and family history of ASD) examined in this study. Analyses of variance were conducted to test the relationship between attitudes/actual knowledge and the categorical variable (i.e., religion and type of ASD for affected child/children). Only the statistically significant variables were then included in the multiple regression analyses that were utilized to evaluate factors associated with parents’ actual knowledge and attitudes pertaining to ASD genetic testing.

All statistical programming was conducted with STATA Version 15.0 with *p* < 0.05 as the criterion for significance. Because the chi-square test is sensitive to large sample sizes [47], construct validity was assessed based on three fit indices: the root mean square error of approximation (RMSEA), comparative fit index (CFI), and standardized root mean residual (SRMR). An RMSEA < 0.08, a CFI > 0.90, and an SRMR < 0.06, were adopted as the cut-off point for an adequate model fit [47,48].

## 3. Results

### 3.1. Demographic Characteristics

Table 1 shows the sociodemographic characteristics of the 443 parents of children diagnosed with ASD who participated in this study. The average age of the participants was 39.9 years (*SD* = 5.4; range = 28–63). The majority of the respondents were female (77.4%), married (88.7%), and employed (61.9%). About two-thirds of the participants (67.2%) had a below-college education level. Furthermore, a large portion of the sample was affiliated with traditional folk religion (29.4%), Buddhism (27.1%), Christianity (9.0%), or more than two religions (13.7%). Most of the participating parents reported no family history of ASD (65.4%), and 94.1% of the participants had only one child diagnosed with ASD. Over half of the participants reported the severity of their child’s ASD as mild (52.9%). Moreover, the child’s type of ASD included autistic disorder (30.5%), Asperger’s syndrome (25.6%), pervasive development disorders not otherwise specified (PDD-NOS) (20.2%), and more than two disorders (5.1%).

### 3.2. Knowledge of ASD Genetic Testing

The mean score of the parents’ actual knowledge of genetic testing for ASD was 2.2 (*SD* = 1.5; range = 0–6) with a theoretical range of 0–7. As shown in Table 2, the average correct rate was 31.4%. None of the participants answered all the questions correctly; a total of 55 participants (13.1%) answered all the questions incorrectly. Moreover, almost all of the participants (*n* = 415, 95.8%) provided incorrect answers for the question about the availability of ASD genetic testing in Taiwan.

Regarding the parents’ perceived knowledge of genetic testing for ASD, the majority of the participants (84.1%) reported that they knew little or very little about ASD genetic testing. Only 10 participants (2.3%) reported that they knew a lot or a great deal about ASD genetic testing; the remaining perceived an average level of knowledge of ASD genetic testing (13.6%).

As presented in Table 3, parents’ actual knowledge of ASD genetic testing was positively associated with their perceived knowledge (B = 0.24, *p* < 0.01) and education level (B = 0.90, *p* < 0.01); it was negatively related to the severity level of their children’s ASD diagnosis (B = −0.27, *p* < 0.01).

### 3.3. Parents’ Attitudes toward ASD Genetic Testing

Table 4 presents the descriptive summary of the parents’ attitudes toward ASD genetic testing. The majority of the participating parents believed that children diagnosed with ASD should undergo ASD genetic testing (66.8%) and agreed that all children with ASD characteristics should undergo ASD genetic testing (64.5%). In addition, most of the participants believed that the biological mother (60.5%) or father (60.9%) of a child with ASD should also undergo ASD genetic testing. However, fewer parents (51.5%) agreed that the siblings of the child with ASD should undergo ASD genetic testing. The items used to examine parents’ attitudes showed a high reliability (Cronbach’s alpha = 0.95) and adequate validity (χ^2^ = 2.14, df = 3, *p* = 0.544, RMSEA = 0, CFI = 1.000, SRMR = 0.002). The sum score of attitude scale was 13.4 (*SD* = 3.30; range = 5–20). The mean score for attitude scale was 2.7 (*SD* = 0.66; range = 1–4).

As presented in Table 5, the multiple regression analysis showed that the parents’ attitudes toward ASD genetic testing were negatively associated with their age (B = −0.07, *p* < 0.05) and education level (B = −0.82, *p* < 0.05). Neither the actual nor perceived knowledge of ASD genetic testing was significantly related to the parents’ attitudes in the binary correlation analyses.

### 3.4. Parents’ Attitudes toward the Cost of ASD Genetic Testing

More than half of the parents (61.9%) either “somewhat agree” or “strongly agree” that undergoing ASD genetic testing is costly for them. The majority of participants (88.1%) believed that the cost of ASD genetic testing should be covered by Taiwanese national health insurance. On average, they were willing to pay NTD 1033.5 (USD ~37.5) for such testing.

## 4. Discussion

Despite the fact that genetic testing for ASD has been available in Taiwan for many years [34,35], little is known about the attitudes toward and knowledge of such testing among Taiwanese parents. To the best of our knowledge, this is the first-of-its-kind survey research to explore parental attitudes and knowledge pertaining to ASD genetic testing in Taiwan. The results of this study suggest that parents of children with ASD in Taiwan generally hold favorable attitudes toward genetic testing for ASD, but they showed deficient knowledge of such testing. This finding is consistent with previous studies exploring parents’ attitudes and knowledge toward ASD genetic testing in the United States and Europe [27,28,29,30,31].

Notably, the participating parents’ attitudes toward ASD genetic testing in this study differed depending on the recipient of the test. Specifically, although the majority of the parents believed that children diagnosed with ASD or with ASD characteristics should undergo ASD genetic testing, only about half of the participants thought the siblings of the child with ASD should also undergo the testing. This finding was different from a study conducted in the United States, in which most of their participants (80%) with a younger undiagnosed child wanted their undiagnosed child tested if it was available [49]. Moreover, in our previous study [50] that assessed Taiwanese parents’ perceived recurrence risk of having another child with ASD, only about half of the participants believed that they had a higher recurrence risk of having another affected child compared to others with normally developing children. Such misperceptions of relative recurrence risk may also influence their attitudes toward genetic testing among the siblings of children diagnosed with ASD [50].

In addition, we also found that the severity level of the children’s ASD was negatively and significantly associated with the parents’ knowledge of genetic testing for ASD. Parents of children with a more severe level of ASD may spend more time and energy on taking care of their children instead of searching for information on ASD genetic testing. Therefore, more targeted health education materials and programs are needed in Taiwan to improve these parents’ awareness and knowledge of ASD genetic testing. These could help the parents have a correct understanding of the ASD genetic testing and potential benefits for siblings of children with a high severity level of ASD.

Interestingly, we found that participating parents with higher education levels showed better actual knowledge of ASD genetic testing but held less favorable attitudes. Besides the potential benefits of ASD genetic testing, parents with higher education levels might have more concerns about the implications of ASD genetic testing, such as privacy and discrimination issues raised by genetic testing [28,51]. This might possibly explain the negative association between parents’ education level and attitudes toward genetic testing for ASD. Moreover, we found that younger parents were more likely to hold positive attitudes toward ASD genetic testing. This finding is in line with previous studies which showed that age was positively correlated with interest in and attitudes toward genetic testing [52,53]. For example, a large survey study conducted in the Netherlands showed that younger respondents exhibited more positive attitudes toward genetic testing compared to older respondents who had a higher likelihood of believing that genetic testing takes away people’s freedoms. Health professionals may consider addressing ASD genetic testing-related concerns and worries raised by older parents and those with higher educational attainment.

Our results showed that the majority of parents in our sample believed that ASD genetic testing was expensive, and most believed that the cost should be covered by national health insurance. If not covered, the average cost that they were willing to pay was NTD 1033.5 (USD~37.5). In Taiwan, however, although national health insurance covers a large number of health services and care [54], it currently does not include ASD genetic testing. ASD genetic testing out-of-pocket expenses range from NTD 18,000 (USD~600) to NTD 30,000 (USD~1000) [35], which is much more expensive than our participants thought was reasonable. Additionally, considering that in Taiwan the average monthly income for each person is USD~2046, the expense of ASD genetic testing is a significant financial burden for parents of children with ASD [35]. Thus, the cost of ASD genetic testing may be a barrier to pursuing this testing for parents of children with ASD. We recommend that the Taiwanese government consider covering ASD genetic testing expenses either entirely or partially through national health insurance for families who have children with ASD in the future.

Our study had some limitations. First, most of the parents in this study were recruited from urban areas of Taiwan, and 77.4% of the participants were mothers. Though mothers are often the main caregivers of children with ASD, future research needs to prioritize the recruitment of more fathers of children with ASD as their attitudes toward genetic testing may be different from those of mothers of children with ASD. Furthermore, similar to other survey research, we were unable to obtain a 100% response rate and study the entire target population [54]. Thus, the findings of this study may not be generalized to all Taiwanese parents of children with ASD as a lack of generalizability is the most common limitation for survey studies [27,30,49]. Second, although the present study examined many sociodemographic characteristics’ associations with parental attitudes and knowledge of ASD genetic testing, other factors (e.g., culture, social norms, as well as perceived benefits and barriers) may still need to be explored to gain a more comprehensive picture of parents’ perspectives. Lastly, this study utilized a correlational approach to assess different factors’ relationships with parental attitudes and knowledge of ASD genetic testing; therefore, causal implications cannot be drawn [54]. However, future research may use our findings to develop tailored education programs and examine their effectiveness in improving parental knowledge and attitudes pertaining to ASD genetic testing.

Despite the limitations, this study provides insights into knowledge of and attitudes towards ASD genetic testing among parents of children with ASD in Taiwan. In line with other studies in the United States and Europe, our findings suggest that parents generally hold positive attitudes toward ASD genetic testing. Nevertheless, most parents lack knowledge on this matter. To improve parental knowledge and awareness regarding ASD genetic testing and assist parents in making informed medical decisions, health education materials and programs on the topic of ASD genetic testing should be developed and implemented in Taiwan. Meanwhile, as physicians are the most important source of information regarding ASD genetic testing for parents in Taiwan [34], more continuing education in this area should be made available to Taiwanese physicians to enhance their knowledge and competency in introducing and educating parents about ASD genetic testing.

## 5. Conclusions

This survey study examined knowledge and attitudes toward ASD genetic testing among parents of children with ASD in Taiwan. Our findings suggest that Taiwanese parents had low levels of knowledge about ASD genetic testing, but generally held positive attitudes regarding such testing. In addition, the majority of our participants believed that ASD genetic testing was currently unaffordable and that national health insurance should cover it. Accordingly, it is important to provide health education to Taiwanese parents of children with ASD to improve their knowledge about ASD genetic testing. We further recommend that future discussions be held to explore the possibility of including ASD genetic testing coverage as a part of national health insurance.

## Figures and Tables

**Table 1:** Sociodemographic characteristics of the sample.

Variable	M (SD) or n	Range or %
Age	39.9 (5.4)	28–63
Gender		
Female	333	77.4%
Male	97	22.6%
Education status		
<College	291	67.2%
≥College	142	32.8%
Marital status		
Married	384	88.7%
Others	49	11.3%
Annual household income		
Less than NTD 20,000 (NTD 600,000)	146	35.2%
NTD 20,000–40,000 (NTD 600,000–1,200,000)	173	41.7%
Above NTD 40,000 (NTD 1,200,000)	96	23.1%
Current employment status		
Employed	268	61.9%
Unemployed	165	38.1%
Religion		
Folk religion	127	29.4%
Buddhism	117	27.1%
Christianity	39	9.0%
More than two religions	59	13.7%
No religion	90	20.8%
Numbers of Children with ASD		
One	415	94.1%
Two	25	5.7%
Three	1	0.2%
Parents with family history of ASD^2^		
No	283	65.4%
Yes	150	34.6%
Severity of the child’s ASD diagnosis		
Mild	222	52.9%
Moderate	129	30.7%
Severe	61	14.5%
Very severe	8	1.9%
Child’s type of ASD		
Asperger’s syndrome	110	25.6%
Autistic disorder	131	30.5%
Pervasive development disorders not otherwise specified (PDD-NOS)	87	20.2%
More than two disorders	22	5.1%
Do not know	80	18.6%

NTD: new Taiwanese dollar; ASD: autism spectrum disorder.

**Table 2:** ASD genetic testing knowledge among parents of children with ASD in Taiwan.

Knowledge Items	Correct (%)
1. Which of the following statements is correct? a. Parents with ASD have higher chance of giving birth to another child with ASD b. ASD is associated with family history c. ASD is associated with genes d. A, B, and C are all correct * e. None of the above is correct f. I don’t know or I am not sure about the answer	46.5%
2. Which of the following statements is correct? a. If a person with ASD marries a normally developed person, they will have 50% chance of having a child with ASD b. If a person with ASD marries another person with ASD, they will have a child with ASD c. If the genetic testing report of a person with ASD suggests that no ASD-associated genes were found, this means that this individual’s ASD has no association with genes d. Genetic testing can definitely find ASD-associated genes. e. None of the above are correct *	41.8%
3. Which of the following groups do not benefit from ASD genetic testing results? a. Children with ASD b. Siblings of a child with ASD c. Biological parents of a child with ASD d. Relatives of a child with ASD e. All of the above groups can benefit from ASD genetic testing results * f. I don’t know or I am not sure about the answer	41.8%
4. Which of the following statement is correct? a. ASD genetic testing is not helpful in treating children with ASD b. ASD genetic testing will not benefit parents of children with ASD c. ASD genetic testing will not benefit children who have been diagnosed with ASD d. A, B, and C are all correct e. None of the above are correct * f. I don’t know or I am not sure of the answer	44.4%
5. Which of the following is correct? a. The ASD genetic testing process is unsafe and risky b. All ASD genetic tests can have over 70% diagnostic yield c. ASD genetic testing can identify the severity level of a child’s ASD d. A, B, and C are all correct e. None of the above is correct * f. I don’t know or I am not sure about the answer	16.0%
6. Which of the following ASD genetic tests are available in Taiwan? a. Fragile X testing b. Chromosomal Microarray Analysis c. ASD genetic testing is still in research and developmental stage. d. Both A and B * e. None of the above f. I don’t know or I am not sure about the answer.	4.2%
7. Which of the following is correct? a. ASD genetic testing can help with the treatment of ASD-related health problems * b. ASD genetic testing can prevent children from having ASD c. ASD genetic testing can prevent the siblings of children with ASD from having ASD d. A, B, and C are all correct e. All of the above are incorrect f. I don’t know or I am not sure about the answer	22.6%
Average	31.4%

* Correct answer.

**Table 3:** Multiple linear regression model of actual knowledge of ASD genetic testing among parents with children of ASD in Taiwan.

Variable	B	β	SE	*t*	Sig. (*p*)
Perceived knowledge	0.24	0.13	0.09	2.78	0.006 **
Participant’s educational level	0.90	0.28	0.17	5.24	0.000 **
Annual household income	0.14	0.07	0.11	1.27	0.204
Family history of ASD	0.17	0.05	0.15	1.09	0.275
Severity of the child’s ASD diagnosis	−0.27	−0.15	0.09	−3.01	0.003 **

** *p* < 0.01.

**Table 4:** Descriptive summary of attitudes toward ASD genetic testing among parents of children with ASD in Taiwan.

Statement	Strongly Agree (%)	Agree (%)	Disagree (%)	Strong Disagree (%)
All children diagnosed with ASD should undergo ASD genetic testing	13.2	53.6	29.0	4.2
All children with ASD characteristics should undergo ASD genetic testing	11.9	52.6	31.4	4.2
All the biological mothers of child with ASD should undergo ASD genetic testing	11.8	48.7	35.3	4.2
All the biological fathers of child with ASD should undergo ASD genetic testing	11.6	49.3	35.4	3.7
All the siblings of child with ASD should undergo ASD genetic testing	9.1	42.5	42.7	5.8

ASD: Autism spectrum disorders. Due to rounding, some percentages do not sum to 100%.

**Table 5:** Multiple linear regression model of attitudes toward ASD genetic testing among parents of children with ASD in Taiwan.

Variable	B	β	SE	*t*	Sig. (*p*)
Participant’s age	−0.07	−0.11	0.03	−2.27	0.024 *
Participant’s educational level	−0.82	−0.12	0.35	−2.38	0.018 *
Participant’s employment status	0.51	0.08	0.33	1.55	0.123

ASD: Autism spectrum disorders; * *p* < 0.05.

## Data Availability

As a data sharing strategy was not included in the original application for institutional review board review, study data are not publicly available.
